# Implications of Pharmacogenetic Testing for Clopidogrel Therapy in a Tertiary Healthcare Hospital in North India

**DOI:** 10.7759/cureus.42169

**Published:** 2023-07-19

**Authors:** Jayachandra Amarapalli, Prabhat Sharma, Rajat Datta, Anuka Sharma

**Affiliations:** 1 Cardiology, Base Hospital, Delhi Cantt, New Delhi, IND; 2 Cardiology, Army Hospital Research and Referral, New Delhi, IND; 3 Cardiology, NMC Genetics Pvt. Ltd., Gurugram, IND

**Keywords:** coronary artery disease (cad), antiplatelet therapy, pharmacogenomics, pci, clopidogrel

## Abstract

Background

Clopidogrel hyporesponsiveness with decreased antiplatelet activity is prevalent in percutaneous coronary intervention (PCI) patients due to reduced function polymorphism in the CYP2C19 enzyme gene which results in poor conversion of this prodrug to an active metabolite. However, pharmacogenetic testing is not part of routine clinical practice in India.

Methodology

In this retrospective observational study, we observed the prevalence of loss of function (LOF) gene variants of CYP2C19 (*2, *3) in 60 patients undergoing PCI with complex anatomies in a tertiary healthcare hospital in North India. We do not have follow-up data for a few patients. However, the treatment regimen was recorded, and the occurrence of any clinical event was monitored for the remaining 52 patients for six months.

Results

The mean age of the patients was 61.76 ± 10.14 years. We found that 52% of patients carried these LOF mutations, of which 37% were intermediate metabolizers, while 15% were poor metabolizers of clopidogrel. However, out of 52 patients for whom follow-up data were available, 22 (42.3%) were intermediate metabolizers, while six (11.54%) showed genotypes associated with poor metabolism of clopidogrel. Clopidogrel (75 mg BD) was the primary replacement drug in place of ticagrelor (90 mg BD) during follow-up after four weeks (based on the clinician’s discretion).

Conclusions

No major ischemic event was reported during the follow-up of these 52 patients. The intermediate metabolizers’ LOF in one copy of the CYP2C19 gene seems to overcome genetic deficiency with the clopidogrel 75 mg BD regime, which is comparable to maintenance with ticagrelor 90 mg BD. This study can be extrapolated to a larger cohort to observe statistically significant differences among various groups.

## Introduction

Nowadays, percutaneous coronary intervention (PCI) is often the choice of treatment for acute coronary syndrome (ACS), and dual antiplatelet therapy (DAPT) with clopidogrel and low-dose aspirin has become the standard of care for maintenance after PCI [1]. Clopidogrel is a prodrug activated in the liver by cytochrome P450 enzyme 2C19 (CYP2C19) and inhibits the platelet P2Y12 receptor, thus attenuating platelet aggregation [2]. CYP2C19 is a highly polymorphic enzyme that results in wide variation in response to clopidogrel in ACS patients [3]. The loss of function (LOF) gene variants of CYP2C19 (*2, *3) are associated with decreased activity of the CYP2C19 enzyme and reduced efficacy of clopidogrel in Asians [4,5]. This association between LOF CYP2C19 gene variants and less platelet inhibition is clinically actionable [5-9]. Alternative antiplatelet drugs (such as ticagrelor or prasugrel), which do not require hepatic bioactivation by the CYP2C19 enzyme, can be considered in such scenarios. However, ticagrelor and prasugrel are associated with a higher risk of bleeding than clopidogrel [3]. Hence, optimal DAPT is a field of debate and ongoing research [10].

This study describes our experiences with genetics-guided clopidogrel therapy in a patient group that was carefully selected with stable coronary artery disease (CAD) (double-vessel disease, triple-vessel disease, restenosis) and the challenges involved in the implementation in a tertiary care hospital in India. Primarily, we focussed on the frequency of clinically actionable genetic test results and outcomes in a resource-constrained Indian scenario.

## Materials and methods

Study design

Patients undergoing PCI at Army Hospital, Delhi Cantt, from May 2018 to February 2020 were included in the study. The Institutional Ethics Committee of Army Hospital, Delhi Cantt, approved the study (approval number: IEC01/April 2022). Our patients were not consecutive CAD patients and were selected based on coronary angiography (CAG). They underwent CAG, and the culprit lesion was addressed, followed by planned PCI for a complex lesion later. A treating cardiologist referred patients, and informed consent was obtained from them. The genetic test results were communicated to the cardiologist, and the subsequent therapy monitoring was done during regular follow-ups at the outpatient department. The operator had a procedural bias based on the complexity of the procedure, and later during follow-up, he wanted to decide whether a patient on clopidogrel should switch to ticagrelor or vice versa. The treatment efficacy was measured by monitoring ischemic events in this observational study.

Genetic analysis

Venous blood samples were collected in ethylenediaminetetraacetic acid-coated vacutainers (BD). According to the manufacturer’s instructions, DNA was extracted using a Qiagen DNA Mini kit in a QIAcube instrument. The quality of extracted DNA was checked using a QIAxpert spectrophotometer (OD 260 nm/280 nm~1.8), and the integrity of purified DNA was verified in 1% agarose gel. The single-nucleotide polymorphisms (SNPs) of CYP2C19 were detected using Sanger Sequencing and Illumina infimum global screening array per manufacturer’s protocol. In our genetic analysis, we included the following CYP2C19-associated SNPs: rs12248560 (*17), rs4244285 (*2), and rs4986893 (*3), which are more prevalent in Asians.

## Results

We included 60 patients undergoing PCI in the study (n = 60). The demographic data of the study population are presented in Table 1.

**Table 1:** Demographic data of the study population. SD = standard deviation; BMI = body mass index

Parameters	n (%)
All patients	60 (100%)
Gender	
Male	47 (78.3%)
Female	13 (21.67%)
Age (years) (mean ± SD)	61.76 ± 10.14
Average BMI (kg/m^2^)	22.85 ± 2.58

The median age of the patients was 61 years (range = 78 to 31 years), and the percentage of female patients was lesser than male patients (21.67% vs. 78.3%, respectively). The results of clopidogrel pharmacogenetic testing are shown in Table 2.

**Table 2:** Results of pharmacogenetic testing in patients (n = 60). *: Indicates gene polymorphism. LOF = loss of function

Phenotype/genotype	n (%)
All patients	60 (100%)
Non-LOF carriers	
Extensive metabolizer	20 (33.33%)
*1/*1	20 (33.33%)
Ultra metabolizer	9 (15%)
*1/*17	9 (15%)
*17/*17	0
LOF carriers	
Intermediate metabolizer	22 (37%)
*1/*2	20 (33.33%)
*1/*3	0
*2/*17	2 (3.33%)
Poor metabolizer	9 (15%)
*2/*2	9 (15%)
*3/*3	0
*2/*3	0

The genetic test results were available four weeks after the patient’s discharge following the PCI procedure. A suitable antiplatelet regimen was selected based on the clinician’s discretion and the patient’s convenience. We found that 52% of patients (n = 31) were carrying alleles with LOF variants, resulting in the limited efficacy of clopidogrel. Among these 31 patients, 22 (37%) were intermediate metabolizers, and nine (15%) were poor metabolizers of clopidogrel. On the other hand, 48% of patients (n = 29) were normal metabolizers of the drug (33.33% extensive metabolizers and 15% ultra metabolizers); therefore, the efficacy of clopidogrel was not reduced in these patients.

Of 60 patients, we have follow-up data for only 52 patients, summarized in Table 3. Overall, 18 (34.6%) patients were extensive metabolizers of clopidogrel. On the other hand, 22 (42.3%) of the 52 patients were intermediate metabolizers of clopidogrel (17 taking ticagrelor 90 mg BD while five patients were taking clopidogrel 75 mg BD). Only six (11.54%) patients among these 52 patients were poor metabolizers of clopidogrel (taking ticagrelor 90 mg BD).

**Table 3:** Treatment regimen of follow-up patients (n = 52).

Phenotype/genotype	n (%)	Treatment regimen
All patients	52 (100%)	
Extensive metabolizer	18 (34.6%)	Clopidogrel 75 mg BD
Intermediate metabolizer	22 (42.3%)	17- ticagrelor 90 mg BD, 5- clopidogrel 75 mg BD
Poor metabolizer	6 (11.54%)	Ticagrelor 90 mg BD

No ischemic events were observed in all 52 patients. However, a noteworthy result was observed in the patient group with clopidogrel intermediate metabolizer genetic profile following two treatment regimens: clopidogrel 75 mg BD and ticagrelor 90 mg BD in six months, as shown in Table 4. No ischemic event was observed in both treatment regimens, indicating their effectiveness in this patient cohort.

**Table 4:** Ischemic events observed in clopidogrel intermediate metabolizer patients with different treatment regimens.

Intermediate metabolizer treatment regimen	Ischemic event observed
Clopidogrel 75 mg BD	Nil
Ticagrelor 90 mg BD	Nil

## Discussion

The decrease in the efficacy of clopidogrel drug due to the presence of the CYP2C19 LOF variant has been demonstrated in various in vitro studies, and its clinical relevance is also established by several good-quality randomized trials [11-17]. Our study identified that 52% of patients had impaired bioactivation of clopidogrel, which is in line with the previously reported prevalence of these variants in the Indian population [18,19]. However, implementing genotype-guided clopidogrel medication is a complex challenge [20]. Many non-genetic factors require a cardiovascular expert’s intervention to decide on a suitable antiplatelet treatment. For example, the latest drug-eluting stents decrease the risk of stent thrombosis with clopidogrel therapy [21]. Additionally, alternative P2Y12 inhibitors such as prasugrel and ticagrelor do not require pharmacogenetic testing. However, the use of prasugrel and ticagrelor is contraindicated in patients with a history of intracranial hemorrhage or ongoing bleeding. Prasugrel is also contraindicated in patients with a body weight less than 60 kg, age over 75 years, renal dysfunction, a history of transient ischemic attack, and those undergoing coronary artery bypass graft surgery [22]. Furthermore, there is poor tolerance in Indian patients for ticagrelor, which is associated with side effects such as dyspnea and shows poor long-term safety. No difference in outcome was observed in our study in clopidogrel intermediate metabolizer patients who switched from ticagrelor 90 mg BD to clopidogrel 75 mg BD. It is difficult for patients to continue with ticagrelor treatment due to its high cost and non-availability in rural areas [23]. Therefore, for pharmacogenomic experts, incorporating such factors into algorithms (generating pharmacogenetic reports for suitable antiplatelet therapy) remains a significant challenge. However, our experience showed that such an algorithm can be developed by collaborating with the genetic testing lab and tertiary care hospital by extrapolating this study to larger CAD patient cohorts and including this testing as a pre-procedure in patients with complex anatomy rather than as an afterthought post-procedure.

## Conclusions

The association between CYP2C19 gene polymorphism and clopidogrel resistance has been intensively explored recently, although no definitive answer has been reached yet. In this retrospective study, we assessed the prevalence of CYP2C19 gene polymorphism and clopidogrel resistance in stable CAD patients with complex anatomies and monitored the treatment outcome. This study is unique in our South Asian Indian population. No such analysis has been performed in this cohort (CAD patients with complex anatomies), and genetic association with phenotype is unique in different people. Moreover, another treatment modality of administering clopidogrel 75 mg BD to the intermediate metabolizers of this drug instead of ticagrelor 90 mg BD is helpful, especially in resource-constrained scenarios, for optimum treatment. Furthermore, this finding can be utilized to direct the usage of clopidogrel in personalized antiplatelet therapy based on genetic testing for better clinical outcomes.
